# Serum sclerostin levels are positively related to bone mineral density in peritoneal dialysis patients: a cross-sectional study

**DOI:** 10.1186/s12882-019-1452-5

**Published:** 2019-07-17

**Authors:** Te-Hui Kuo, Wei-Hung Lin, Jo-Yen Chao, An-Bang Wu, Chin-Chung Tseng, Yu-Tzu Chang, Hung-Hsiang Liou, Ming-Cheng Wang

**Affiliations:** 1 Division of Nephrology, Department of Internal Medicine, National Cheng Kung University Hospital, College of Medicine, National Cheng Kung University, 138 Shengli Rd., North Dist, Tainan, 704 Taiwan; 2 Department and Graduate Institute of Public Health, College of Medicine, National Cheng Kung University, Tainan, Taiwan; 3 Department of Internal Medicine, National Cheng Kung University Hospital, College of Medicine, National Cheng Kung University, Tainan, Taiwan; 4 Institute of Clinical Pharmacy and Pharmaceutical Sciences, College of Medicine, National Cheng Kung University, Tainan, Taiwan; 5 Division of Nephrology, Department of Internal Medicine, Hsin-Jen Hospital, 395 Zhongzheng Rd., Xinzhuang Dist, New Taipei City, 242 Taiwan

**Keywords:** Sclerostin, Bone mineral density, Peritoneal dialysis, Chronic kidney disease–mineral and bone disorder (CKD–MBD), Wnt pathway

## Abstract

**Background:**

Sclerostin, an antagonist of the Wingless-type mouse mammary tumor virus integration site (Wnt) pathway that regulates bone metabolism, is a potential contributor of chronic kidney disease (CKD)–mineral and bone disorder (MBD), which has various forms of presentation, from osteoporosis to vascular calcification. The positive association of sclerostin with bone mineral density (BMD) has been demonstrated in CKD and hemodialysis (HD) patients but not in peritoneal dialysis (PD) patients. This study assessed the association between sclerostin and BMD in PD patients.

**Methods:**

Eighty-nine PD patients were enrolled; their sera were collected for measurement of sclerostin and other CKD–MBD-related markers. BMD was also assessed simultaneously. We examined the relationship between sclerostin and each parameter through Spearman correlation analysis and by comparing group data between patients with above- and below-median sclerostin levels. Univariate and multiple logistic regression models were employed to define the most predictive of sclerostin levels in the above-median category.

**Results:**

Bivariate analysis revealed that sclerostin was correlated with spine BMD (*r* = 0.271, *P* = 0.011), spine BMD T-score (*r* = 0.274, *P* = 0.010), spine BMD Z-score (*r* = 0.237, *P* = 0.027), and intact parathyroid hormone (PTH; *r* = − 0.357, *P* < 0.001) after adjustments for age and sex. High BMD, old age, male sex, increased weight and height, diabetes, and high osteocalcin and uric acid levels were observed in patients with high serum sclerostin levels and an inverse relation was noticed between PTH and sclerostin. Univariate logistic regression analysis demonstrated that BMD is positively correlated with above-median sclerostin levels (odds ratio [OR] = 65.61, *P* = 0.002); the correlation was retained even after multivariate adjustment (OR = 121.5, *P* = 0.007).

**Conclusions:**

For the first time, this study demonstrated a positive association between serum sclerostin levels and BMD in the PD population.

## Background

Chronic kidney disease (CKD)–mineral and bone disorder (MBD) is a systemic disorder caused by alterations in mineral handling, bone and hormonal changes due to CKD, and subsequent cardiovascular disease and mortality; CKD–MBD becomes a potential target to improve CKD care outcomes referred by the KDIGO CKD–MBD guidelines [1]. CKD–MBD is characterized by a disruption in the homeostasis of the renal, skeletal, and cardiovascular systems. The injured kidney contributes to hyperphosphatemia, excessive bone resorption, and subsequent osteopenia in CKD patients with either high- or low-turnover bone disorders resulting in osteoporosis prevalence being higher in patients with CKD than in the general population [2–4]. By accelerating ectopic mineralization, particularly vascular calcification, CKD–MBD extends beyond CKD-related bone disorders toward increasing cardiovascular risk and mortality by further aggravating vascular stiffness and promoting ventricular hypertrophy development. Over the past decade, numerous studies have reported that the wingless-type mouse mammary tumor virus integration site (Wnt)/β-catenin canonical signaling pathway is a major bone mass regulatory pathway [5]. Wnt signaling is mediated by the binding of Wnt ligands to a transmembrane receptor complex, composed of the frizzled receptor and either of the two coreceptors, the low-density lipoprotein receptor-related protein (LRP) 5 or LRP6 [6]. Wnt signaling activation reduces osteoblast and osteocyte apoptosis, induces osteoblastogenesis, and inhibits osteoclastogenesis [7]. These actions, resulting in subsequent increase in bone formation and bone density, may be crucial in CKD–MBD pathogenesis [8].

The Wnt signaling pathway is regulated by several inhibitors, whose regulatory action involves either preventing Wnt ligands from binding to receptors directly or binding to LRP5 or LRP6 (thus reducing the availability of these coreceptors to Wnt ligands). Of the main regulators in the Wnt pathway, dickkopf (DKK)-1 and particularly sclerostin have recently been studied comprehensively. DKK-1, a 28-kDa glycoprotein found in osteoblasts and osteocytes, inhibits the Wnt pathway by binding to LRP5, LRP6, and Kremen-1 (another coreceptor) to induce internalization of these coreceptors [9]. Similar to DKK-1, sclerostin is a 24-kDa glycoprotein, encoded by *SOST*, but secreted almost exclusively by osteocytes. Sclerostin inhibits the Wnt pathway by competing with Wnts for binding to LRP5 and LRP6 to antagonize the canonical Wnt signaling. In studies mainly on non-CKD population, sclerostin levels were correlated positively with age, male sex, diabetes, and bone mineral density (BMD) but inversely with physical activity and parathyroid hormone (PTH) levels [10–14].

Patients with CKD have increased bone loss and low BMD risks followed by fractures because of the disturbance in mineral and bone metabolism [15, 16]. Recent CKD-MBD guidelines have updated that BMD assessment is sufficient for fracture prediction in patients with advanced CKD [17]. However, most studies correlating sclerostin with BMD and other CKD–MBD-related factors mainly involve nondialyzed CKD or hemodialysis (HD) patients and very few have been in the peritoneal dialysis (PD) population [18–22]. Thus, this study explored serum sclerostin levels and their association with BMD and other CKD–MBD-related biomarkers in the PD patients.

## Methods

### Patients

This study enrolled patients with end-stage renal disease receiving PD for at least 3 months at the National Cheng Kung University Hospital, a tertiary hospital in Taiwan. The patients attended PD care follow-ups at monthly intervals. A written informed consent was obtained from each patient before study participation. The study protocol was approved by the Institutional Review Board of National Cheng Kung University Hospital (approval number: B-ER-103-279), according to the Ethics of Clinical Research, Declaration of Helsinki.

### Biochemistry and clinical parameters

For blood examination, all samples were collected and sent to the central laboratory of the hospital in the index month for analysis. The blood examination included complete blood cell count and serum albumin, blood urea nitrogen, creatinine, aspartate transaminase (AST), alanine transaminase (ALT), sodium, potassium, calcium, phosphorus, magnesium, alkaline phosphatase (ALP), uric acid, bicarbonate, total cholesterol, triglyceride, high-density lipoprotein cholesterol (HDL), low-density lipoprotein cholesterol (LDL), iron profiles, high-sensitivity C-reactive protein (hsCRP), and intact PTH measurement.

At the same time, serum samples were collected and stored in a refrigerator at − 80 °C for further analyses. Other non-PD care–related examination but related to CKD–MBD included the measurement of serum levels of sclerostin, fibroblast growth factor (FGF)-23, DKK-1, and crosslinked C-telopeptide of type 1 collagen (CTX) through enzyme-linked immunosorbent assay (ELISA). Moreover, osteocalcin and 25(OH)-vitamin D levels (sent to the central laboratory of the hospital) were measured, along with pulse wave velocity (VaSera VS-1000; Fukuda Denshi, Tokyo, Japan), ankle brachial index (VaSera VS-1000; Fukuda Denshi, Tokyo, Japan), BMD examination, and X-ray examination of pelvis and bilateral hands (for peripheral vascular calcification scoring [23]) were also performed in the same month.

### ELISA for sclerostin, FGF-23, DKK-1, and CTX measurements

Serum sclerostin, FGF-23, DKK-1, and CTX levels were measured using commercial quantitative sandwich ELISA kits by Biomedica (Vienna, Austria; assay limit: 3.2 pmol/L, coefficients of variation (CV): 3–10%, median for healthy individuals: 24.14 pmol/L), Kainos (Tokyo, Japan; assay limit: 3 pg/mL, CV < 10%, mean for healthy individuals: 42.0 pg/mL), R&D Systems (Minneapolis, USA; assay limit: 4.2 pg/mL, CV: 3.3–7.6%, mean for healthy individuals: 2513 pg/mL), and Cloud-Clone Corp. (Wuhan, China; assay limit: 53.4 pg/mL, CV < 10%, range for healthy individuals: 0.45–2.4 ng/mL), respectively.

### Bone mineral densitometry

Anteroposterior lumbar spine (L1–L4) BMD measurement (in g/cm^2^) was performed through dual-energy X-ray absorptiometry by using a LUNAR Prodigy Model (GE Healthcare, Madison, USA), according to the standard protocol in the manufacturer’s instructions. For quality assurance of BMD measurement, certified densitometry operators implemented a quality assurance program by including a daily calibration measurement and using standard phantoms supplied with the system, according to the manufacturer’s instructions. The mean CV for lumbar spine in our center is 0.25%. The total lumbar spine BMD was calculated by averaging the BMD of L1–L4 vertebrae. BMD T and Z scores were classified according to World Health Organization criteria.

### Statistical analyses

Baseline demographics and comorbidities are reported as means ± standard deviations (SD), medians and interquartile ranges (IQR), or number and percentages, as appropriate. Spearman rank correlation coefficients were used to examine the relationship between serum sclerostin levels and each clinical parameter. Partial correlation coefficients were assessed after age and gender adjustment. To perform further analysis, we stratified patients evenly into two groups for comparison according to median serum sclerostin levels because the normal serum sclerostin range in the PD population remains unclear. Grouped data were compared through Student *t*, Mann–Whitney U, or chi-square testing, as appropriate. All relevant variables with statistical significance in the grouped data comparison were tested through univariate logistic regression modeling to define the most predictive of sclerostin levels in the above-median category. Multiple logistic regression analysis was subsequently performed to assess the contribution of the clinical and laboratory parameters, with adjustments for age, sex, and weight; according to the sample size estimates, as maximum of four predictors can be included in multiple regression for the 89 samples. Box-Tidwell testing was performed to examine whether the explanatory continuous variables comply with the linearity assumption of logistic regression in the logit [24]. The data were analyzed on SAS (version 9.4 for Windows; SAS Institute Inc., Cary, NC, USA), with *P* < 0.05 being considered statistically significant.

## Results

### Patient characteristics

In total, 89 PD patients were enrolled. Table 1 lists their demographic data, comorbidities, and blood examination and image study results: age, 49.2 ± 10.6 years; male-to-female ratio, 0.49; PD vintage, 1490 (649–2392) days; body mass index, 23.6 ± 3.8; total Kt/V for urea (i.e., peritoneal Kt/V plus residual renal Kt/V), 1.95 (1.84–2.09); number of anuric patients, 46 (51.7%); and serum sclerostin level, 80.2 (60.2–107.0) pmol/L. Approximately 19.1% of the patients had received parathyroidectomy, and only 14.6% had diabetes. Mild femoral vascular calcification was noted in the X-ray of only four patients, and no patient demonstrated bilateral hand vascular calcification. Because ankle brachial index and pulse wave velocity data were available for further analysis, we have not presented the vascular calcification scores here.

**Table 1:** Patient characteristics and comparison between patients with above- and below-median sclerostin levels

Characteristics	Total	Sclerostin ≥ 80.2 pmol/L	Sclerostin < 80.2 pmol/L	*P* value
Number of patients	89	45	44	
Age (year)	49.2 ± 10.6	51.4 ± 10.3	47.0 ± 10.6	0.048
Sex-male	44 (49.4)	28 (62.2)	16 (36.4)	0.015
Dialysis vintage (day)	1490 (649–2392)	1401 (742–2385)	1777 (552–2431)	0.752
Weight (kg)	62.5 ± 13.0	65.7 ± 12.3	59.2 ± 13.0	0.016
Height (cm)	162.4 ± 8.1	164.1 ± 7.4	160.7 ± 8.6	0.048
BMI (kg/m^2^)	23.6 ± 3.8	24.3 ± 3.7	22.8 ± 3.8	0.051
Total Kt/V for urea	1.95 (1.84–2.09)	1.94 (1.82–2.08)	1.97 (1.86–2.10)	0.036
Baseline comorbidities				
Diabetes mellitus	13 (14.6)	10 (22.2)	3 (6.8)	0.040
Hypertension	62 (69.7)	33 (73.3)	29 (65.9)	0.446
Parathyroidectomy	17 (19.1)	10 (22.2)	7 (15.9)	0.449
Anuria	46 (51.7)	24 (53.3)	22 (50.0)	0.753
Serum biochemistry				
Sclerostin (pmol/L)	80.2 (60.2–107)	107.0 (87.0–135)	69.1 (50.6–69.1)	< 0.001
FGF-23 (pg/mL)	3193 (1878–3547)	3193 (2003–3442)	3173(1863–3559)	0.902
DKK-1 (pg/mL)	981 ± 356	1048 ± 402	912 ± 290	0.071
CTX (ng/mL)	2.70 (2.14–3.38)	2.68 (2.19–3.25)	2.91 (2.08–3.66)	0.408
25(OH)-Vitamin D (ng/mL)	22.7 ± 7.5	24.2 ± 7.3	21.3 ± 7.4	0.067
Osteocalcin (ng/mL)	177 (98.3–349)	137 (97.3–264)	228 (106–563)	0.025
Intact PTH (pg/mL)	315 (119–564)	199 (111–458)	399 (169–718)	0.008
Urea nitrogen (mg/dL)	64.7 ± 17.2	65.9 ± 16.4	63.5 ± 18.1	0.520
Creatinine (mg/dL)	12.6 ± 2.9	13.3 ± 2.6	12.0 ± 3.0	0.038
Calcium (mg/dL)	9.6 ± 0.7	9.5 ± 0.7	9.7 ± 0.7	0.345
Phosphorus (mg/dL)	5.1 (4.3–6.0)	5.1 (4.3–6.0)	5.1 (4.2–6.2)	0.735
Magnesium (mg/dL)	2.1 ± 0.4	2.1 ± 0.4	2.1 ± 0.4	0.994
Albumin (g/dL)	3.9 (3.7–4.1)	3.9 (3.7–4.2)	3.9 (3.7–4.1)	0.999
Alkaline phosphatase (U/L)	104 (70–143)	92 (68–120)	115 (71–159.5)	0.191
Cholesterol (mg/dL)	186.0 ± 44.6	183.4 ± 41.4	188.7 ± 48.0	0.581
Triglyceride (mg/dL)	141 (104–254)	155 (88–257)	132 (104–224)	0.620
Hemoglobin (g/dL)	10.5 (9.6–11.4)	10.8 (9.8–11.6)	10.4 (9.4–10.9)	0.062
Glucose (mg/dL)	96 (91–113)	101 (92–122)	93 (89–99.5)	0.012
Uric acid (mg/dL)	6.8 ± 1.4	7.1 ± 1.4	6.4 ± 1.3	0.021
hsCRP (mg/L)	2.36 (0.81–8.97)	3.53 (0.89–8.97)	2.14 (0.54–7.91)	0.548
BMD, spine L1 to L4 (g/cm^2^)	1.16 ± 0.18	1.22 ± 0.17	1.10 ± 0.18	0.001
BMD spine T-score	0.12 ± 1.50	0.56 ± 1.40	−0.33 ± 1.47	0.004
BMD spine Z-score	0.32 ± 1.32	0.74 ± 1.31	− 0.12 ± 1.19	0.002
Ankle brachial index	1.10 ± 0.09	1.09 ± 0.10	1.11 ± 0.08	0.352
Pulse wave velocity (m/s)	15.0 ± 2.5	15.1 ± 2.7	14.9 ± 2.2	0.783

*BMI* body mass index, *FGF-23* fibroblast growth factor 23, *DKK-1* dickkopf-1, *CTX* cross-linked C-telopeptide of type 1 collagen, *PTH* parathyroid hormone, *hsCRP* high-sensitivity C-reactive protein, *BMD* bone mineral density

### Correlations between serum sclerostin level and clinical parameters

Bivariate analysis (Table 2) revealed that sclerostin was positively associated with male sex (*r* = 0.266, *P* = 0.011); weight (*r* = 0.210, *P* = 0.048); height (*r* = 0.256, *P* = 0.016); serum 25(OH)-vitamin D (*r* = 0.235, *P* = 0.026), creatinine (*r* = 0.295, *P* = 0.005), hemoglobin (*r* = 0.286, *P* = 0.007), and glucose (*r* = 0.287, *P* = 0.007) levels; spine BMD (*r* = 0.277, *P* = 0.009); and spine BMD T (*r* = 0.237, *P* = 0.025) and Z (*r* = 0.232, *P* = 0.029) scores. By contrast, it was inversely associated with intact PTH levels (*r* = − 0.300, *P* = 0.004). The relationship of sclerostin with creatinine (*r* = 0.262, *P* = 0.014), hemoglobin (*r* = 0.257, *P* = 0.016), and glucose (*r* = 0.291, *P* = 0.006) levels; spine BMD (*r* = 0.271, *P* = 0.011); spine BMD T (*r* = 0.274, *P* = 0.010) and Z (*r* = 0.237, *P* = 0.027) scores; and intact PTH (*r* = − 0.357, *P* < 0.001) remained significant after adjustment for age and sex.

**Table 2:** Bivariate correlation between sclerostin levels and clinical parameters

Parameters	r_s_	*P* value	adjusted r_s_	*P* value
Age (year)	0.108	0.314		
Sex-male	0.266	0.011		
Dialysis vintage (day)	0.068	0.528	0.149	0.167
Weight (kg)	0.210	0.048	0.087	0.427
Height (cm)	0.256	0.016	0.101	0.359
Body mass index (kg/m^2^)	0.108	0.312	0.060	0.580
Total Kt/V for urea	−0.072	0.505	0.010	0.926
Diabetes mellitus	0.185	0.083	0.179	0.098
Hypertension	0.089	0.407	0.091	0.402
Parathyroidectomy	0.056	0.601	0.104	0.339
Anuria	0.120	0.261	0.165	0.126
FGF-23 (pg/mL)	−0.066	0.540	−0.127	0.242
DKK-1 (pg/mL)	0.171	0.109	0.174	0.107
CTX (ng/mL)	−0.081	0.453	−0.098	0.369
25(OH)-vitamin D (ng/mL)	0.235	0.026	0.131	0.227
Osteocalcin (ng/mL)	−0.168	0.116	−0.191	0.076
Intact PTH (pg/mL)	−0.300	0.004	−0.357	< 0.001
Urea nitrogen (mg/dL)	0.074	0.492	0.054	0.620
Creatinine (mg/dL)	0.295	0.005	0.262	0.014
Calcium (mg/dL)	−0.091	0.396	− 0.110	0.309
Phosphorus (mg/dL)	−0.003	0.981	−0.017	0.876
Magnesium (mg/dL)	−0.022	0.841	−0.025	0.816
Albumin (g/dL)	0.013	0.905	−0.031	0.776
Alkaline phosphatase (U/L)	−0.201	0.059	−0.214	0.047
Cholesterol (mg/dL)	−0.183	0.086	−0.126	0.244
Triglyceride (mg/dL)	0.010	0.927	0.047	0.664
Hemoglobin (g/dL)	0.286	0.007	0.257	0.016
Glucose (mg/dL)	0.287	0.007	0.291	0.006
Uric acid (mg/dL)	0.180	0.091	0.183	0.089
hsCRP (mg/L)	−0.004	0.972	0.002	0.982
BMD, spine L1 to L4 (g/cm^2^)	0.277	0.009	0.271	0.011
BMD spine T-score	0.237	0.025	0.274	0.010
BMD spine Z-score	0.232	0.029	0.237	0.027
Ankle brachial index	−0.095	0.377	−0.170	0.119
Pulse wave velocity (m/s)	0.031	0.776	0.067	0.537

*r*_*s*_ Spearman rank correlation coefficient, *adjusted r*_*s*_ partial correlation coefficient adjusted for age and gender, *BMI* body mass index, *FGF-23* fibroblast growth factor 23, *DKK-1* dickkopf-1, *CTX* crosslinked C-telopeptide of type 1 collagen, *PTH* parathyroid hormone, *hsCRP* high-sensitivity C-reactive protein, *BMD* bone mineral density

### Differences in patient characteristics according to serum sclerostin levels

The characteristics of patients with above- and below-median serum sclerostin levels are compared in the right columns of Table 1. Sclerostin levels were associated positively with age, male sex, weight, height, diabetes, BMD, and serum uric acid levels and negatively with total Kt/V for urea, osteocalcin levels, and intact PTH levels. No association between arterial stiffness and sclerostin was observed.

### Logistic regression for factors associated with above-median sclerostin levels

Univariate analysis (Table 3) revealed a significant correlation of sclerostin with male sex (odds ratio [OR] = 2.882, *P* = 0.016), uric acid (OR = 1.454, *P* = 0.025), and BMD (OR = 65.607, *P* = 0.002). Age, weight, diabetes, osteocalcin levels, and intact PTH levels had marginally statistical significance (*P* > 0.05) or marginal effects (OR = ~ 1) on sclerostin levels. Multiple logistic regression results indicated that osteocalcin (OR = 0.998, *P* = 0.016), intact PTH (OR = 0.998, *P* = 0.006), and uric acid (OR = 1.481, *P* = 0.034) levels and BMD (OR = 121.5, *P* = 0.007) were correlated to high serum sclerostin levels with statistical significance; however, some of the effects remained marginal. No continuous predictor demonstrated nonlinearity with statistical significance in the Box-Tidwell testing for the logistic regression.

**Table 3:** Logistic regression analysis of association of above-median sclerostin levels with clinical and biochemical parameters

Variable	Univariate analysis	*P* value	Multivariate analysis			
			Model 1^a^	*P* value	Model 2^b^	*P* value
	Odds ratio (95% CI)		Odds ratio (95% CI)		Odds ratio (95% CI)	
Age (year)	1.042 (1.000–1.087)	0.052				
Sex-male	2.882 (1.219–6.815)	0.016				
Weight (Kg)	1.041 (1.003–1.081)	0.035	1.030 (0.988–1.074)	0.167		
Total Kt/V for urea	0.311 (0.045–2.145)	0.236	0.560 (0.071–4.447)	0.584	0.414 (0.041–4.190)	0.455
Diabetes mellitus	3.905 (0.995–15.317)	0.051	3.757 (0.932–15.141)	0.063	3.183 (0.685–14.791)	0.140
Osteocalcin (ng/mL)	0.998 (0.997–1.000)	0.034	0.998 (0.997–1.000)	0.027	0.998 (0.996–1.000)	0.016
Intact PTH (pg/mL)	0.998 (0.997–1.000)	0.015	0.998 (0.996–0.999)	0.009	0.998 (0.996–0.999)	0.006
Uric acid (mg/dL)	1.454 (1.049–2.016)	0.025	1.511 (1.068–2.137)	0.020	1.481 (1.030–2.129)	0.034
BMD, spine L1 to L4 (g/cm^2^)	65.61 (4.414–975)	0.002	172.0 (6.675–4431)	0.002	121.5 (3.634–4061)	0.007
BMD spine T-score	1.546 (1.129–2.117)	0.007	1.862 (1.261–2.749)	0.002	1.792 (1.173–2.737)	0.007
BMD spine Z-score	1.783 (1.209–2.630)	0.001	1.838 (1.203–2.806)	0.005	1.765 (1.143–2.724)	0.010

*CI* confidence interval, *PTH* parathyroid hormone, *BMD* bone mineral density

^a^model 1: adjusted for age and gender; ^b^model 2: adjusted for age, gender, and weight

## Discussion

Our findings demonstrated, for the first time in a PD population, that sclerostin levels are positively associated with BMD, an association that remained even after adjustment for various factors. Because gender and weight can have different effects on BMD, their contribution to the sclerostin–BMD association was also investigated. In the general population, particularly postmenopausal women, a positive correlation between BMD and sclerostin has been identified [11, 25–27]. This relationship can also be observed in older men and patients with advanced CKD, including those receiving maintenance HD [11, 21, 28, 29]. However, this positive association between sclerostin and BMD cannot be well explained by the BMD-lowering effects of sclerostin, which was demonstrated by an interventional study, in which a lower risk of vertebral fractures was noted in postmenopausal women with osteoporosis after antagonizing sclerostin [30]. In addition, high physical activity, particularly weight-bearing activity, aided in reducing sclerostin levels and thus increased BMD [31]. In another study on healthy men, long-term bed rest reduced BMD and increased sclerostin levels [32]. These results disagree with the assumption that serum sclerostin levels increase and thus BMD decreases in older people and CKD or dialysis patients because their physical activity levels typically are low. Nevertheless, considering the causal inferences in this association from the opposite direction, a possible explanation could be higher BMD in men and patients with higher weights reflect higher total body skeletal mass with more osteocytes, the main sclerostin-producing cells [11].

Sclerostin levels also increase with age in nondialyzed CKD and HD patients [18, 33]. In 2015, Yamada et al. reported that serum sclerostin was positively associated with age in PD patients [34]. However, in the present study, patients with above-median sclerostin levels were characterized by older age, with only marginally statistical significance, probably because of our limited sample size.

Moreover, here, intact PTH levels were negatively associated with serum sclerostin levels (Spearman correlation coefficient adjusted for age and gender, *r* = − 0.357, *P* < 0.001). Intact PTH is a significant determinant of serum sclerostin levels in healthy premenopausal and postmenopausal women [10, 35], patients with primary hyperparathyroidism [14], HD patients [8], and PD patients [34], but not in patients with non-dialyzed CKD [18]. Although the PTH–sclerostin relationship was seen in our univariate and multivariate analyses, the effect size was marginal. Some of our patients had received parathyroidectomy, which may interfere with their intact PTH levels; thus, whether parathyroidectomy has a role in circulating sclerostin remains unclear. Because further subgroup analysis was limited by the sample size of our parathyroidectomy patients, we performed another analysis to test for the intact PTH–parathyroidectomy interaction for serum sclerostin levels; however, no significant interaction was noted (P for interaction = 0.440). Patients with a parathyroidectomy history are mostly present in the dialysis population; two studies have explored sclerostin levels in these dialysis patients [21, 34]. However, patients with parathyroidectomy were excluded by Cejka et al. (when including HD patients) and by Yamada et al. (when including PD patients). Hence, more data and studies to elucidate the relationship between serum intact PTH and sclerostin levels after parathyroidectomy in these dialysis patients are warranted.

An unexpected finding in the present study was the uric acid–sclerostin relationship. In the univariate logistic regression, uric acid was positively correlated with sclerostin, with statistical significance. In the multiple regression analysis with adjustments for age, gender, and weight, this association remained significant (*P* < 0.05). Thus far, no direct link between uric acid and sclerostin has been reported; nevertheless, more data to clarify this association are needed.

Sclerostin is also positively associated with pulse wave velocity for arterial stiffness in postmenopausal women, nondialyzed CKD patients, HD patients, and kidney transplant recipients and thus could be a biomarker for the cardiovascular changes in the CKD–MBD spectrum [22, 25, 36, 37]. However, in our PD patients, such an association was absent between arterial stiffness and sclerostin. Although the results were nonsignificant in our PD patients, vascular stiffness could be a later presentation after sclerostin or BMD has changed. The evolving disturbances in the renal–skeletal–cardiovascular axis in the CKD–MBD may target osteoporosis with an early intervention to prevent further cardiovascular damage and improve long-term outcomes; thus, in the early CKD–MBD phase, sclerostin could both be a biomarker and an intervention target. However, we did not obtain this kind of association between arterial stiffness and sclerostin in the PD patients.

In addition, to explore the relationship between BMD and the bone-related biomarkers other than sclerostin, we compared the respective associations (Table 4). The relationship between DKK-1 and BMD disclosed inconsistent results in previous reports: DKK-1 had a negative correlation with BMD in diabetes and predialysis CKD patients [28, 38], but in postmenopausal women, HD patients, and people under steroid treatment, this relationship was not observed [21, 25, 39]; moreover, here, we did not find this correlation in our PD patients. Although both sclerostin and DKK-1 express Wnt antagonism, they are independent (not synergistic) regulators of Wnt signaling. DKK-1 and sclerostin do not bind simultaneously to LRP5; DKK-1 can displace sclerostin from a previously formed sclerostin-LRP5 complex [40]. In addition, compared with the BMD-sclerostin association, the lack of BMD-DKK-1 association may be related to DKK-1 not being highly expressed in adult bones unless it is activated by an insult. By contrast, sclerostin expression is downregulated when bone formation or normal skeletal maintenance is required, not merely when fractures occur [41]. The differences in study populations and the wider tissue distribution of DKK-1 compared with that of sclerostin may explain the varying results of their correlation with BMD [25]. FGF-23 is positively correlated significantly with BMD in older men and postmenopausal women [42–44]. However, the association was not noted in the CKD or HD patients [45, 46]. After bone degradation, CTX, a bone resorption marker, is released into circulation and eventually excreted through urine [47]. CTX is negatively correlated with BMD in HD patients, but not PD patients [48, 49]—similar to our results. Because CTX is renally cleared, residual renal function may alter sclerostin levels. Here, we analyzed CTX results by stratifying our PD patients into anuric and nonanuric groups but observed no difference in CTX levels or total Kt/V between these two groups. According to a study on CTX in HD patients, CTX was nonsignificantly associated with the residual renal function, and CTX sampling time was suggested to be before dialysis because the concentration decreased after HD, implying some HD-related clearance [50]. Osteocalcin, produced by osteoblasts, is often considered a bone formation marker. Our results revealed that BMD is negatively correlated with osteocalcin after adjustments for age and gender; similar results were reported in predialysis CKD and HD patients [51, 52]. Nevertheless, here, the correlation between bone-related biomarkers other than sclerostin and BMD was not strong; however, the trends of the associations in this study were compatible with most of the previous reports. Further information enabling the elucidation of the bone-related biomarker relationships with BMD in PD patients is needed because the actual association may be merely masked by either our small sample size or our inclusion of younger patients with narrow age distribution.

**Table 4:** Bivariate correlation between bone-related biomarkers and spine BMD

	BMD, spine L1 to L4				BMD, spine T-score				BMD, spine Z-score			
Parameters	r_s_	*P* value	adjusted r_s_	*P* value	r_s_	*P* value	adjusted r_s_	*P* value	r_s_	P value	adjusted r_s_	*P* value
Age (year)	−0.225	0.034			−0.245	0.021			0.124	0.248		
Sex-male	0.184	0.085			0.006	0.955			−0.017	0.877		
Sclerostin (pmol/L)	0.277	0.009	0.271	0.011	0.237	0.025	0.274	0.010	0.232	0.029	0.237	0.027
FGF-23 (pg/mL)	0.124	0.248	0.049	0.650	0.093	0.386	0.060	0.579	−0.018	0.866	0.004	0.969
DKK-1 (pg/mL)	0.017	0.876	−0.022	0.838	0.020	0.852	−0.008	0.937	−0.101	0.347	−0.087	0.423
CTX (ng/mL)	−0.080	0.455	−0.051	0.642	−0.087	0.419	−0.055	0.610	−0.118	0.272	−0.137	0.205
Osteocalcin (ng/mL)	−0.199	0.062	−0.273	0.010	−0.202	0.057	−0.254	0.017	−0.352	< 0.001	−0.339	0.001
Intact PTH (pg/mL)	−0.192	0.072	−0.242	0.024	−0.202	0.058	−0.220	0.041	−0.291	0.006	−0.291	0.006
Alkaline phosphatase (U/L)	−0.201	0.059	−0.120	0.268	−0.184	0.085	−0.120	0.270	−0.150	0.160	−0.201	0.062

*r*_*s*_ Spearman rank correlation coefficient, *adjusted r*_*s*_ partial correlation coefficient adjusted for age and gender, *FGF-23* fibroblast growth factor 23; *DKK-1* dickkopf-1, *CTX* crosslinked C-telopeptide of type 1 collagen, *PTH* parathyroid hormone, *BMD* bone mineral density

Although our results demonstrated the sclerostin–BMD association, our clinical inference should be interpreted with caution because the selection of site for BMD measurement and quantification of sclerostin with different assays may demonstrate important differences. Lumbar BMD may increase if abdominal aortic calcification or spinal degeneration exists [53–56]. Here, the review of pelvis and previous abdominal X-ray revealed that few of our enrolled patients had aorta calcification or severely degenerative spine, which could strongly influence spine BMD accuracy. Although BMD measurement is recommended by the KDIGO 2017 Clinical Practice Guideline Update [17], the choice of site for BMD measurement is not clearly stated and recommended. Four prospective studies of BMD and incident fractures in adults were reviewed in guideline development process. The purpose was for fracture prediction and the BMD results of hip or femoral neck were with a higher rate of prediction. However, a few dialysis patients receive total hip replacement surgery because the long-term use of steroid for glomerulonephritis in their predialysis phase has side effects and BMD at these sites cannot be measured. Therefore, in addition to fracture risk assessment, lumbar BMD may still have a role for the assessment of CKD-MBD syndrome, but the evaluation of factors affecting the accuracy of BMD measurement should be considered simultaneously. Regarding sclerostin measurement, Delanaye et al. reported that commercially available sclerostin ELISAs demonstrated marked differences in the values of measurement [57]. These differences potentially result from the manufacturer-provided antibodies being different, having crossreactivity with other proteins, and capturing both intact sclerostin and its degraded fragments. In the present study, ELISA for sclerostin may have detected both the intact molecule and its fragments; however, no information on the activity of degraded fragments of sclerostin is currently available. When exploring the relationship between sclerostin and its clinical or biochemical determinants, our results should be used with caution until a reference assay with high specificity for sclerostin is available.

This study has some limitations. First, similar to any cross-sectional observational study, our study confirmed the expected association, but a causal inference could not be made. To characterize the sclerostin–BMD relationship, a future longitudinal study may be needed. Second, our sample was relatively small and thus its statistical power may not have been sufficient for detecting potential confounding factors affecting sclerostin levels. Third, the biological variation in the serum levels of sclerostin and other bone-related biomarkers may cause some bias in a single measurement for each patient. Fourth, only lumbar spine BMD was measured and the correlation between sclerostin and BMD at other sites in PD patients was not investigated. In addition, we did not obtain information on the physical activities, which could be BMD determinants; however, dialysis patients are typically thought to have relatively low weight-bearing activities. Fifth, we did not collect peritoneal dialysate for sclerostin measurement and thus whether the clearance of sclerostin through peritoneal dialysate potentially interfered with our final result remains unclear. Nevertheless, the total Kt/V for urea in our patients, as a surrogate of sclerostin clearance through peritoneal dialysate, demonstrated no major effects for sclerostin in our analysis. Finally, sclerostin levels are considerably influenced by the assay used; our findings would not be immediately relevant if a different assay were adopted.

## Conclusions

This is the first study demonstrating that serum sclerostin is significantly correlated with BMD in PD patients. The BMD of vertebrae was positively associated with sclerostin levels even after adjustment for multiple factors. The results add substantial evidence to the whole picture of CKD–MBD by connecting sclerostin to bone changes in the CKD–MBD spectrum. However, the detailed effects of sclerostin from the skeletal to vascular sides of the CKD–MBD spectrum remain unclear. Thus, future longitudinal studies elucidating the role of sclerostin in PD patients with CKD–MBD are warranted.

## Data Availability

All data supporting the study are presented in the manuscript and available on a request to either of the corresponding authors of this manuscript, Ming-Cheng Wang. or Hung-Hsiang Liou.

## Abbreviations

ALP: Alkaline phosphatase
ALT: Alanine transaminase
AST: Aspartate transaminase
BMD: Bone mineral density
CKD: Chronic kidney disease
CKD–MBD: Chronic kidney disease–mineral and bone disorder
CTX: Crosslinked C-telopeptide of type 1 collagen
DKK-1: Dickkopf-1
ELISA: Enzyme-linked immunosorbent assay
FGF-23: Fibroblast growth factor 23
HD: Hemodialysis
HDL: High-density lipoprotein cholesterol
hsCRP: High-sensitivity C-reactive protein
IQR: Interquartile range
LDL: Low-density lipoprotein cholesterol
LRP: Low-density lipoprotein receptor-related protein
OR: Odds ratio
PD: Peritoneal dialysis
PTH: Parathyroid hormone
SD: Standard deviation
Wnt: Wingless-type mouse mammary tumor virus integration site
